# Exploratory Factor Analysis for Validating Traditional Chinese Syndrome Patterns of Chronic Atrophic Gastritis

**DOI:** 10.1155/2016/6872890

**Published:** 2016-12-19

**Authors:** Yin Zhang, Ajian Zhou, Yue Liu, Ying Zhao, Li Zhang, Leilei Sun, Shiyu Du, Qiang Yang, Xin Song, Chaoyang Liang, Xia Ding

**Affiliations:** ^1^Beijing University of Chinese Medicine, Chaoyang District, Beijing 100029, China; ^2^Institute of Basic Research in Clinical Medicine, China Academy of Chinese Medical Sciences, Dongcheng District, Beijing 100700, China; ^3^School of Humanities, Beijing University of Chinese Medicine, Chaoyang District, Beijing 100029, China; ^4^Institute of Traditional Chinese Medicine, Beijing University of Chinese Medicine, Chaoyang District, Beijing 100029, China; ^5^Department of Neurosurgery, China-Japan Friendship Hospital, Chaoyang District, Beijing 100029, China; ^6^Institute of Systems Engineering, Faculty of Management and Economics, Dalian University of Technology, Ganjingzi District, Dalian 116024, China; ^7^Department of Gastroenterology, China-Japan Friendship Hospital, Beijing 100029, China; ^8^Department of Pathology, China-Japan Friendship Hospital, Chaoyang District, Beijing 100029, China; ^9^Department of General Surgery, China-Japan Friendship Hospital, Chaoyang District, Beijing 100029, China; ^10^Department of Thoracic Surgery, China-Japan Friendship Hospital, Chaoyang District, Beijing 100029, China

## Abstract

*Background.* Traditional Chinese medicine (TCM) has long been used to treat chronic atrophic gastritis (CAG). The aim of the present study was to evaluate the TCM syndrome characteristics of CAG and its core pathogenesis so as to promote optimization of treatment strategies.* Methods.* This study was based on a participant survey conducted in 4 hospitals in China. Patients diagnosed with CAG were recruited by simple random sampling. Exploratory factor analysis (EFA) was conducted on syndrome extraction.* Results.* Common factors extracted were assigned to six syndrome patterns:* qi* deficiency,* qi* stagnation, blood stasis, phlegm turbidity, heat, and* yang* deficiency. Distribution frequency of all syndrome patterns showed that* qi *deficiency,* qi *stagnation, blood stasis, phlegm turbidity, and heat excess were higher (76.7%–84.2%) compared with* yang* deficiency (42.5%). Distribution of main syndrome patterns showed that frequencies of* qi* deficiency,* qi* stagnation, phlegm turbidity, heat, and* yang* deficiency were higher (15.8%–20.8%) compared with blood stasis (8.3%).* Conclusions.* The core pathogenesis of CAG is combination of* qi* deficiency,* qi* stagnation, blood stasis, phlegm turbidity, heat, and* yang *deficiency. Therefore, treatment strategy of herbal prescriptions for CAG should include herbs that regulate* qi*, activate blood, resolve turbidity, clear heat, remove toxin, and warm yang.

## 1. Background

Chronic atrophic gastritis (CAG) is an inflammatory disease of the stomach from various etiologies [1–3]. Typical symptoms, when present, include epigastric pain, fullness, belching, anorexia, and other nonspecific symptoms [3]. CAG can lead to mucosal atrophy, intestinal metaplasia (IM), and gastric intraepithelial dysplasia (GED), also known as intraepithelial neoplasia, which is defined as the precancerous stage of gastric carcinoma [1, 4, 5]. Global cancer statistics for 2012 estimated that there were 951,600 new cases of stomach cancer worldwide [6]. The transition from chronic gastritis to gastric cancer is a typical disease model of uncontrolled inflammation leading to malignant transformation [7–10]. Active treatment of CAG arrests further malignant transformation, and thus prevents gastric cancer [11, 12].

Traditional Chinese medicine (TCM) has a long history in treating gastritis. Numerous basic and clinical studies have demonstrated that Chinese medicine can effectively treat CAG, including resolving mucosal inflammation and reversing glandular atrophy, as well as inhibiting or even reversing intestinal metaplasia and gastric epithelial dysplasia [13–24]. Modern-day TCM treatment of CAG remains based on the time-honored core principle of syndrome pattern differentiation to identify and treat the root of illness. When differentiating a patient's syndrome pattern, the TCM practitioner systematically collects comprehensive information about the presenting signs and symptoms, using the four diagnostic methods of looking, listening/smelling, asking, and palpating. The collocated information is then evaluated according to TCM theory and clinical experience to identify the physical condition and nature of pathologic changes during the current stage of the disease. Treatment is then applied in accordance with conclusions drawn from the pattern differentiation process.

Factor analysis (FA) is a statistical analytic method for reducing data, that is, the redundancy of variables, and to detect the structure (relationships) among the variables being measured. FA has been applied in TCM to develop usable dimensional taxonomies, by which large numbers of observed variables can be remodeled as linear combinations of a smaller number of the underlying factors. There are two modes of FA. Exploratory factor analysis (EFA) is an exploratory data-driven tool that generates solutions for developing theories. Its aim is to explore the relationships among the variables without a specific hypothesis or a priori fixed number of factors. Confirmatory factor analysis (CFA) is a method for theory testing that requires the researcher to have substantive knowledge and a firm idea about the number of factors that will be come upon during analysis. In the field of TCM, EFA is being used increasingly for data mining of measured variables such as clinical information obtained during syndrome pattern differentiation. Through EFA features of the distribution of syndromes can be ascertained.

Although various studies have demonstrated that TCM can effectively treat CAG [13–24], there has been a lack of consensus across studies on syndrome differentiation, treatment strategy, and elaboration of herbal prescriptions [25–27]. This is likely due to the complexity of CAG pathogenesis in TCM. Results of literature reviews have confirmed the lack of agreement in CAG syndrome features [28–33] with only a few studies focusing mainly on CAG. This situation is not conducive to standardization of syndrome differentiation and improvement in treatment efficacy of CAG. The present study explored syndrome pattern features and core TCM pathogenesis of CAG by applying EFA to provide evidence for establishment of TCM treatment principles and standardization of syndrome differentiation for chronic atrophic gastritis.

## 2. Methods

### 2.1. Study Design and Participants

This study was conducted between September 2010 and October 2012 based on participant survey. Outpatients and inpatients who visited the hospital for upper gastrointestinal endoscopy and were diagnosed with CAG were recruited by simple random sampling from medical centers in Beijing and Xiamen, China: Dongzhimen Hospital Affiliated to Beijing University of Chinese Medicine, Peking Union Medical College Hospital Affiliated to the Chinese Academy of Medical Sciences, Beijing Hospital Affiliated to the National Health and Family Planning Commission of China, and Xiamen Traditional Chinese Medicine Hospital. All participants signed consent forms. Ethics approval of the present study was given by the Medical Ethics Committee of Beijing University of Chinese Medicine.

### 2.2. Diagnostic Criteria

Diagnostic criteria of CAG were based on Chinese Society of Gastroenterology, Chinese Medical Association, guidelines [1].

### 2.3. Inclusion and Exclusion Criteria

Inclusion criteria were as follows: (1) meeting the diagnostic criteria of CAG with detailed medical records and diagnostic reports, (2) age: 20–75 years old, (3) willing to participate in the investigation and sign informed consent, and (4) capable of completing the clinical observation questionnaire and responding to the investigator queries.

Exclusion criteria were as follows: (1) unclear diagnosis or not meeting the diagnostic criteria of CAG, (2) not meeting the age criteria; (3) cognitive difficulty such that four diagnostic methods cannot be completed accurately; (4) other digestive disease or neurologic, circulatory, respiratory, and endocrine disease.

### 2.4. Case Report Form Content and Administration

Content of the CAG case report form (CRF) was based on literature research, on expert advice, and on standard Chinese guidelines [34–37]. CRF content included general information (name, sex, and age), disease data (complaint, history of present illness, past medical history, family medical history, and endoscopy results), information from the four diagnoses (symptoms, physical signs, tongue appearance, and pulse reading), western medicine diagnosis, and TCM diagnosis.

Disease history and results of the four diagnostic methods were collected and recorded. Records that met the following criteria were deemed acceptable: all general information filled in except address and contact details and complete information for CAG and four diagnostic methods.

The study was carried out with strict quality control. All investigators were specialized in TCM or integrated Chinese and Western medicines and trained in standard operating procedures of the study. Each study participant was examined and followed up by at least two resident physicians or graduate students who filled in the CRFs. At least two senior staff physicians supervised the interview sessions to ensure consistency and authenticity of data collection to reduce measurement bias.

### 2.5. Data Analysis

**Figure 1:**
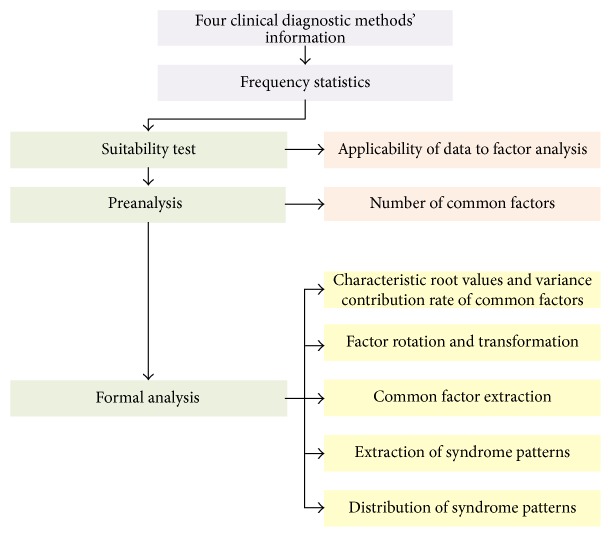
Flow chart of EFA based CAG syndrome patterns extraction.

Frequency analysis was performed on data collected from the four diagnostic methods. Exploratory factor analysis (EFA) was performed on syndrome element extraction (Figure 1). All statistical analyses in this study were processed by SPSS software (version 17.0, SPSS Inc., Chicago, IL, USA).

## 3. Results

### 3.1. Characteristics of Participants

A total of 135 CRFs were distributed, and 131 were completed and returned, with a return rate of 97%. There were 120 forms, that is, patients, that were deemed eligible for the study after eliminating questionnaires with incomplete information, thus, with the acceptance rate of 92%. Among the participants, 62 were male and 58 were female, with an average age of 52.56 and a standard deviation of 12.45.

### 3.2. Distribution Characteristics of Results from the Four Diagnostic Methods

Information from the four diagnostic methods was collected and 53 entries with ≥10% frequency were tabulated based on the distribution frequency (Table 1).

Data were preanalyzed and ultimately 40 diagnostic variables with more than 21% frequency of occurrence were chosen to judge the applicability of the data for EFA, so as to determine the number of common factors to be extracted for formal analysis.

### 3.3. Suitability Test

KMO and Bartlett's test of sphericity were used to evaluate suitability of collocated diagnostic variables for EFA. The Kaiser-Meyer-Olkin (KMO) test assesses partial correlation between variables, and if the KMO value is >0.5, the variable will be more suitable for EFA. In addition, the closer the KMO value is to 1, the stronger the correlation is between variables. Bartlett's test of sphericity assesses the null hypothesis and whether the correlation matrix is rejected as a unit matrix. Only when variables are relatively nonindependent (*P* < 0.05) can they be used for EFA.

In this study, the KMO value of the partial correlation of variables was 0.549 > 0.5 (Table 2), indicating a certain degree of partial correlations between variables, such that EFA could be carried out. The approximate chi-square value of Bartlett's test of sphericity was 1589.24 and *P* < 0.001, indicating strong correlation between variables, rejection of the hypothesis of independence of variables, and that the variables could be applied to EFA.

### 3.4. Assessment of Common Factors

Characteristic root value is an index to evaluate the influence of extracted common factors; that is, introduction of this common factor can explain and evaluate the information of the original variables. Variance contribution rate is the proportion of communality in all variances, and the value is positively correlated with carrying capacity of comprehensive information. The cumulative variance contribution rate is the accumulation of variance contribution rate of first N common factors, which represents the proportion of information of all variables whose first N common factors is covered. Results of characteristic root values and variance contribution rate of common factors were tabulated (Table 3). Principal component analysis was applied to extract common factors. Characteristic root values of the first 15 common factors were greater than 1, and their cumulative variance contribution rates reached 70.795, good overall data interpretation capability.

**Figure 2:**
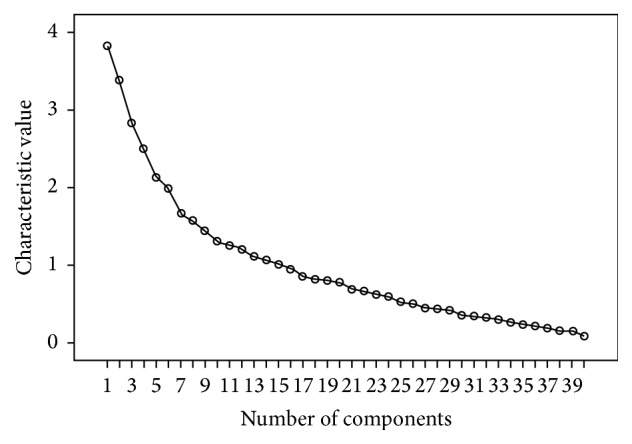
Scree plot of characteristic root value of common factors.

Scree plot displayed relevance of common factors and characteristic root values (Figure 2). The number of common factors was shown on the *x*-axis and the characteristic values on the *y*-axis. Scatter locations of first 15 common factors were steep, and characteristic root values of the last 25 common factors were small as revealed by the leveling off of the curve's slope. Thus, the number of extracted common factors in formal calculation was 15.

### 3.5. Factor Rotation and Transformation

Factor rotation was performed to allow the factor load absolute value of the new common factor for each of the four diagnostic results to polarize to 0 or 1 and thus more clearly display the load of all common factor variables, as well as maintaining the variance of all common factors in the corresponding row unchanged. This allows for a more reasonable explanation for the extraction of all common factors. Principal component analysis was used to extract 15 common factors, and the factor rotation method used was varimax rotation. Rotation was converged after 20 iterations and results of the factor load matrix after rotation transformation is shown in Table 4.

### 3.6. Common Factor Extraction

In Table 4, the factor load value was the coefficient values of each common factor that were used to reflect the closeness between common factors and variables. The essence of factor load values was the correlation coefficient between them. A positive factor load value from the four diagnostic methods' information represents a positive correlation, and a negative factor load value represents a negative correlation. Correlation between them had a positive correlation with factor load value. Load coefficients that were positive and larger or equal to 0.25 of the four diagnostic methods' information were selected into the corresponding factors.

Through consultation with TCM gastroenterology experts throughout China, the diagnostic variables, nature of disease, and disease location obtained from the four diagnostic methods' information were assigned to 15 common factors (F) (Table 5).

### 3.7. Extraction of Syndrome Patterns

Next, the 15 common factors were combined based on syndrome pattern and organ location. Ultimately, six syndrome patterns were established (Table 6).

### 3.8. Distribution of Syndrome Patterns

**Figure 3:**
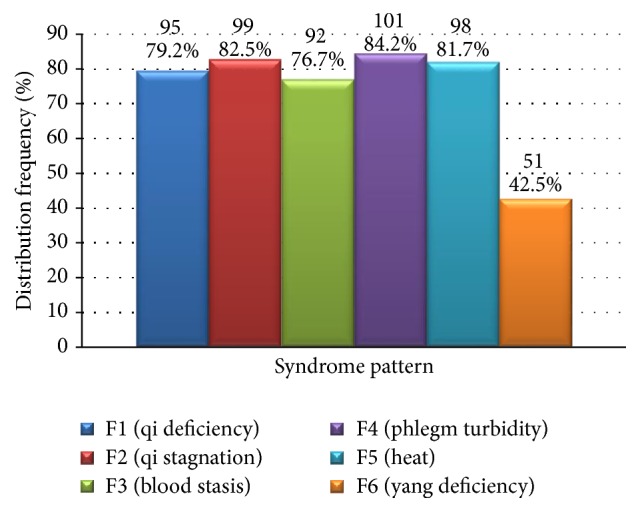
Distribution of all syndrome patterns (*N* = 120).

The corresponding common factor score of each participant's six syndrome patterns was calculated based on the factor score coefficient, according to which all syndrome patterns of each participant were estimated. Distribution of all syndrome patterns was tabulated (Figure 3).

**Figure 4:**
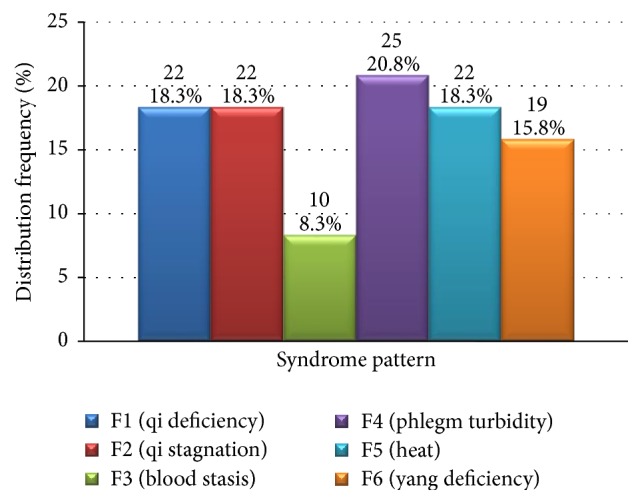
Distribution of primary syndrome patterns (*N* = 120).

Factor load after rotation and transformation yielded a syndrome pattern with the highest score, which was taken as the primary syndrome pattern. Distribution of primary syndrome patterns was shown in Figure 4.

## 4. Discussion

EFA is a multivariate statistical analytic method to explain original variable correlation with potential variables from the perspective of original variable correlation information. The fundamental concept is to project high-dimensional information onto a lower plain to explore internal structure and essential characteristics through dimensionality reduction. EFA applies principal component analysis to extract common factors. Constant factor variance and difference maximization (relative load of squares) are achieved by factor rotation. Finally, complex original data set information is summarized to a limited number of unmeasurable latent variables (common factors) to describe information of most variables. Based on this, the relationship where original measured variables are governed by a small number of independent factors is explored, and the nature of the original variables is clarified by a linear combination of common factors [38–42].

The holistic theory that TCM is based on determines the complex multivariate nonlinear, that is, multicollinear, relationship of the variables of the four diagnostic methods' information. In this study using EFA to assess TCM syndrome characteristics of CAG the four diagnostic variables were the original variables and had significantly higher dimensional characteristics. The syndrome patterns and other syndrome information were extracted from the common factors (four diagnostic variables with specific combination of features), which were the governing correlations of the four diagnostic variables, so as to achieve dimensionality reduction and elimination of multicollinearity.

The four diagnostic methods' information that were detected with high frequency were further assessed to rule out any noise interference after frequency statistics. EFA was applied to extract the syndrome patterns, organ location, and other common factors related to the four diagnostic methods' information. All information the common factors belonged to was appraised based on professional knowledge to extract the syndrome patterns. The distribution features of all syndrome patterns and their corresponding primary syndrome patterns were determined. Thus, EFA showed a total of 15 extracted common factors comprised 6 syndrome patterns (*qi* deficiency,* qi* stagnation, blood stasis, phlegm turbidity, heat, and yang deficiency) as well as disease location in the liver, spleen, or stomach.

In TCM, the syndrome pattern is the basic pathogenetic unit for evaluating a disease [43, 44]. Extraction of the aforementioned 6 syndrome patterns suggests that they form the core pathogenesis of CAG and should thus be the fundamental diagnostic elements taken into account during differentiation of CAG. It follows that, when formulating a prescription to treat CAG, the 6 strategies that should be considered depending on the presenting syndrome pattern are regulating* qi* (tonification of* qi* and moving of* qi*), activating blood, resolving turbidity, clearing heat, removing toxin, and warming yang. Furthermore, target organs of treatment should be the liver, spleen, and stomach.

In this study, EFA results indicated that the syndrome pattern blood stasis was highly associated with* qi* deficiency,* qi* stagnation, phlegm turbidity, and heat, whereas association of* yang* deficiency with the same syndrome patterns was significantly lower. However, in terms of distribution of the primary syndrome pattern, the detected frequency was significantly lower than that of* qi* deficiency,* qi* stagnation, phlegm turbidity, blood stasis, and yang deficiency. Thus, from two seemingly contradictory results, it can be inferred that blood stasis, similar to* qi* deficiency,* qi* stagnation, phlegm turbidity, and heat, has a wide distribution as a fundamental syndrome pattern and has a high frequency of detection even in patients who have mild blood stasis. For this reason, of all the syndrome patterns, blood stasis had the lowest distribution frequency. This suggests that, in terms of treatment of CAG, formulation of prescriptions should focus on the combination of tonification and moving of* qi*, activating blood and resolving stasis, clearing phlegm to resolve turbidity, clearing heat and removing toxin, and activating blood to resolve stasis. However, herbs that activate blood to resolve stasis should be prescribed in lesser amounts.

Interestingly, analysis of all syndrome patterns showed that frequency of* yang* deficiency was significantly lower than that of* qi* deficiency,* qi *stagnation, phlegm turbidity, and heat. EFA of primary syndrome patterns indicated that distribution frequency of* yang* deficiency was similar to that of* qi* deficiency,* qi* stagnation, phlegm turbidity, and heat, and was significantly higher than that of blood stasis. Thus, unlike* qi* deficiency,* qi* stagnation, phlegm turbidity, and heat, yang deficiency was not widely detected in CAG patients as a fundamental syndrome pattern. Although overall distribution was narrow, once* yang* deficiency took hold in the body, its presence was intense and detectable, thus occupying a considerable proportion of the primary syndrome distribution. Therefore, in formulating a prescription, in the early stage of* yang* deficiency type CAG, herbs that tonify* yang* should receive less prominence than herbs that focus on tonifying and regulating* qi*, activating blood, resolving phlegm turbidity, clearing heat, and removing toxin. If* yang* deficiency persists,* yang*-tonifying herbs should be used to some extent.

Other investigations have not been consistent on TCM syndrome patterns and disease site of CAG [28–32]. For example, Gan and colleagues' [28] conclusion regarding disease site (stomach, spleen, and liver) concurred with our findings. However, their results on primary syndrome patterns of CAG (combined deficiency and excess and combined cold and heat) differed from our results of six patterns. Literature reviews have also confirmed the lack of agreement among TCM authors regarding disease site and primary syndrome patterns of CAG [30, 31], indicating the complexities of both the disease nature and progression of CAG as well as the differentiation of its syndrome patterns. Thus, our study applied EFA in an attempt to describe the network of relationships among the four diagnostic variables in order to understand the main syndrome patterns and thus disease sites of CAG.

There are some limitations of this study. Selection bias may exist because all data were derived from participants in hospitals in only two cities with a relatively small sample and therefore are likely not representative of the four diagnostic variables in CAG patients in the rest of China. Future multicentered study which includes large samples is required to verify the conclusions of the present study. In terms of data mining methodology, in EFA each variable or common factor has only one chance to be included in a single category, which means multiple correspondence between variables and categories cannot be created. These flaws may result in an inability to accurately describe the internal property or external relevance of TCM variables from a multidimensional and multilinear perspective to some extent. So in this process, multicollinearity is eliminated by reducing the dimension, from which information may be lost by the so-called dimension effect. It should be noted that Bartlett's test of sphericity approximate chi-square value of EFA was 1589.243 (*P* < 0.001) and the KMO value was 0.549 > 0.5, indicating that EFA was acceptable. However, the degree of perfection was not satisfactory such that the results need to be validated with other supervised data mining methods.

## 5. Conclusions

EFA is a valuable methodology for developing usable dimensional taxonomies in TCM, by which observed syndrome-related variables can be remodeled as linear combinations of the underlying factors. Application of EFA can provide evidence for treatment principles and standardization of syndrome differentiation of chronic atrophic gastritis. Results of this study found that the core pathogenesis of CAG is a combination of* qi* deficiency,* qi* stagnation, blood stasis, phlegm turbidity, and heat and yang deficiency. TCM treatment of CAG should therefore focus on regulating* qi*, activating blood, resolving turbidity, clearing heat and removing toxin, and warming yang.

## Figures and Tables

**Table 1:** Four diagnostic variables' frequency for chronic atrophic gastritis (*N* = 120).

Frequency sequence	Four diagnostic variables	Distribution frequency (%)
1	Wiry pulse	75.8
2	Dark tongue	65
3	Distention and fullness of the stomach	64.2
4	Thin tongue coating	61.7
5	White tongue coating	59.2
6	Greasy tongue coating	56.7
7	Belching	54.2
8	Stomach aversion to cold	54.2
9	Acid regurgitation	50
10	Lack of strength	50
11	Dry mouth	49.2
12	Heartburn	46.7
13	Irritability	45
14	Bitter taste in the mouth	45
15	Yellow tongue coating	44.2
16	Red tongue	39.2
17	Insomnia	35.8
18	Pale red tongue	34.2
19	Loose stool	33.3
20	Profuse dreaming at night	33.3
21	Constipation	32.5
22	Enlarged tongue	32.5
23	Sensation of coldness of the body and limbs	31.7
24	Fetid mouth odor	30
25	Choppy pulse	30
26	Stifling sensation in the chest	28.3
27	Poor appetite	28.3
28	Night sweat	28.3
29	Thick tongue coating	28.3
30	Frequent sighing	25.8
31	Abdominal distention	24.2
32	Palpitations	23.3
33	Listlessness	23.3
34	Teeth-marked tongue	23.3
35	Fine pulse	23.3
36	Tinnitus	22.5
37	Sharp pain in stomach	21.8
38	Nausea	21.8
39	Tongue with petechiae	21.8
40	Slippery pulse	21.8
41	Sallow complexion	21.8
42	Hypochondriac pain	20.8
43	Dry stool	20.8
44	Hiccups	20.8
45	Rapid pulse	20.8
46	Distending pain in the stomach	20
47	Stomach noises (grumbling)	20
48	Thirst and desire to drink	20
49	Emotional upset	19.2
50	Reddish yellow urine	19.2
51	Feeling of heaviness of the body	19.2
52	Dull ache in the stomach	18.3
53	Distention and fullness in the hypochondriac region	16.7
54	Dark complexion	16.7
55	Shortness of breath	15.8
56	Borborygmus	15.8
57	Thin and shrunken tongue	15
58	Sticky mouth	13.3
59	Flatulence	12.5
60	Sunken pulse	12.5
61	Bland taste in the mouth	10.8

**Table 2:** KMO and Bartlett's test of sphericity.

Kaiser-Meyer-Olkin measure of sampling adequacy	0.549

Bartlett's test of sphericity	
Approx. chi-square	1589.24
df	780
Sig	0.000

**Table 3:** Characteristic root values and variance contribution rate of common factors.

Common factor	Initial characteristic root value			Variance extraction load			Variance load after rotation		
	Characteristic root value	Variance contribution rate (%)	Cumulative variance contribution rate (%)	Characteristic root value	Variance contribution rate (%)	Cumulative variance contribution rate (%)	Characteristic root value	Variance contribution rate (%)	Cumulative variance contribution rate (%)
1	3.830	9.576	9.576	3.830	9.576	9.576	1.988	4.970	4.970
2	3.382	8.454	18.030	3.382	8.454	18.030	1.903	4.756	9.726
3	2.832	7.080	25.110	2.832	7.080	25.110	1.107	2.768	12.494
4	2.507	6.268	31.378	2.507	6.268	31.378	1.035	2.588	15.082
5	2.132	5.330	36.708	2.132	5.330	36.708	1.028	2.570	17.651
6	1.987	4.967	41.675	1.987	4.967	41.675	1.027	2.568	20.219
7	1.671	4.176	45.852	1.671	4.176	45.852	1.027	2.567	22.786
8	1.575	3.937	49.789	1.575	3.937	49.789	1.026	2.566	25.352
9	1.443	3.608	53.396	1.443	3.608	53.396	1.025	2.564	27.915
10	1.296	3.239	56.636	1.296	3.239	56.636	1.025	2.562	30.478
11	1.262	3.155	59.791	1.262	3.155	59.791	1.022	2.556	33.034
12	1.218	3.044	62.836	1.218	3.044	62.836	1.018	2.546	35.580
13	1.106	2.765	65.601	1.106	2.765	65.601	1.018	2.546	38.126
14	1.055	2.638	68.239	1.055	2.638	68.239	1.018	2.544	40.669
15	1.022	2.556	70.795	1.022	2.556	70.795	1.017	2.542	43.212
16	.956	2.391	73.186	.956	2.391	73.186	1.017	2.542	45.754
17	.852	2.130	75.316	.852	2.130	75.316	1.017	2.541	48.295
18	.810	2.025	77.341	.810	2.025	77.341	1.014	2.536	50.831
19	.805	2.013	79.354	.805	2.013	79.354	1.014	2.534	53.365
20	.774	1.934	81.288	.774	1.934	81.288	1.011	2.527	55.892
21	.696	1.740	83.028	.696	1.740	83.028	1.008	2.520	58.412
22	.666	1.665	84.693	.666	1.665	84.693	1.007	2.517	60.929
23	.624	1.560	86.253	.624	1.560	86.253	1.005	2.512	63.441
24	.591	1.476	87.729	.591	1.476	87.729	1.004	2.509	65.950
25	.526	1.315	89.044	.526	1.315	89.044	1.002	2.504	68.455
26	.495	1.239	90.283	.495	1.239	90.283	1.001	2.502	70.957
27	.451	1.127	91.410	.451	1.127	91.410	1.001	2.502	73.459
28	.437	1.093	92.503	.437	1.093	92.503	.997	2.493	75.952
29	.421	1.053	93.555	.421	1.053	93.555	.997	2.492	78.444
30	.351	.878	94.433	.351	.878	94.433	.993	2.482	80.926
31	.336	.839	95.272	.336	.839	95.272	.981	2.453	83.379
32	.323	.807	96.079	.323	.807	96.079	.975	2.439	85.817
33	.294	.734	96.813	.294	.734	96.813	.965	2.413	88.230
34	.257	.643	97.456	.257	.643	97.456	.959	2.397	90.627
35	.236	.589	98.044	.236	.589	98.044	.935	2.337	92.964
36	.212	.529	98.574	.212	.529	98.574	.907	2.267	95.232
37	.194	.484	99.058	.194	.484	99.058	.863	2.159	97.390
38	.157	.392	99.451	.157	.392	99.451	.767	1.918	99.308
39	.139	.348	99.798	.139	.348	99.798	.184	.459	99.767
40	.081	.202	100.000	.081	.202	100.000	.093	.233	100.000

*Note.* Extraction method: principal component analysis. Rotation method: Kaiser standardized orthogonal. Rotation was converged after 20 iterations.

**Table 4:** Factor load matrix after rotation transformation.

Four diagnostic variables	Component														
	1	2	3	4	5	6	7	8	9	10	11	12	13	14	15
Distention and fullness of the stomach	−.028	.005	.015	.056	**.332**	−.026	.121	.024	.160	**.694**	.093	−.202	.008	−.053	−.009
Palpitations	**.544**	.021	.195	.136	.051	.242	.167	−.057	.046	.026	.029	−.130	**−.321**	**.359**	−.109
Stifling sensation in the chest	.140	.020	.136	.017	.139	.010	.096	−.019	−.091	.034	.041	−.021	.056	**.833**	.072
Loose stool	−.073	.128	−.047	**.416**	.113	−.086	.209	−.075	**.449**	−.049	.180	.068	.027	.034	.192
Stomach tingling Sharp pain in stomach	−.095	−.170	**.356**	.048	−.014	.201	**.478**	.214	.041	.185	−.060	.101	**−.341**	−.076	.213
Frequent sighing	**.629**	**−.281**	.050	−.060	−.121	−.100	.058	.046	.016	.035	.074	**.251**	.080	.119	.215
Constipation	−.205	−.081	.089	**.502**	.145	.026	−.038	−.195	.133	**.324**	.142	.141	**.363**	−.105	.142
Irritability	.017	−.024	.248	.013	−.118	**.590**	.164	.079	−.132	**−.327**	.136	−.095	.161	−.141	.101
Bitter taste in the mouth	.047	−.010	.049	.133	**.282**	**.622**	−.074	−.156	−.056	.094	−.200	.024	.036	−.061	.195
Poor appetite	−.050	.013	−.037	.087	**−.253**	.095	−.097	−.065	−.158	**.720**	−.014	.008	.024	.064	.035
Abdominal distention	.060	−.009	−.027	.142	.101	.235	.195	−.051	.187	.187	.114	.200	**.295**	.124	**.414**
Fetid mouth odor	.078	.041	−.075	−.034	.165	.044	.078	.064	−.007	−.020	−.054	−.033	−.054	.039	**.814**
Belching	.142	.009	−.192	.074	**.425**	−.027	.065	.016	.018	.172	.131	**.458**	−.180	.051	.017
Sallow complexion	.029	.041	.029	.005	−.057	.005	.116	−.039	**.775**	−.024	.007	.010	.032	−.092	−.046
Acid regurgitation	.012	−.098	−.097	.149	**.794**	.075	.006	.010	.006	−.112	.027	.041	.050	.131	.143
Stomach aversion to cold	**.670**	.150	−.146	.000	.208	.074	−.074	.011	.068	−.139	−.093	.216	−.064	.008	.056
Heartburn	.065	−.203	.152	−.143	**.702**	.183	.126	−.158	−.052	.106	−.059	−.004	.090	.013	.070
Listlessness	**.388**	−.002	−.032	**.730**	.024	−.021	.066	.159	.093	−.035	−.059	.126	−.175	.040	−.092
Sensation of coldness of the body and limbs	**.795**	.169	−.088	.125	.068	−.043	.052	.058	−.047	.022	−.012	−.096	.191	−.017	−.055
Nausea	.174	.056	.028	**.325**	.064	−.181	.050	.213	−.004	−.061	−.270	**.462**	**.363**	**.395**	.021
Lack of strength	−.011	.010	.038	**.738**	−.005	.190	−.033	.098	−.047	.166	.014	**−.318**	−.044	.037	−.024
Insomnia	−.163	−.054	−.079	−.081	.161	**.561**	−.129	.088	**.426**	.035	**.353**	−.082	.168	.130	−.072
Profuse dreaming at night	.110	−.112	.020	−.112	.062	**.349**	−.112	.004	.068	.040	.027	−.071	**.786**	.051	−.031
Dry mouth	.066	.063	.204	.070	.102	**.569**	.081	−.068	−.014	**.341**	−.129	.173	.161	.175	−.086
Tinnitus	**.395**	.082	−.018	**.316**	−.135	.032	.345	−.127	**−.315**	−.035	.180	.100	.131	.163	.127
Pale red tongue	.042	−.122	−.040	.105	−.117	.045	−.073	**.860**	.023	.038	.035	.061	.051	−.081	.011
Red tongue	.015	−.006	**.386**	−.015	−.031	.182	−.034	**−.724**	.017	.175	−.131	−.012	.111	−.133	−.068
Dark tongue	.099	.170	−.160	−.082	.088	−.097	**.768**	−.033	−.046	−.190	.045	−.019	.046	.044	.237
Enlarged tongue	.100	.160	−.122	**.312**	−.126	−.018	−.083	**.251**	**.479**	.014	**−.226**	−.138	.016	.000	**.293**
Tongue with petechiae	−.013	−.069	−.057	.079	.056	.083	**.669**	−.058	**.266**	.180	−.110	.061	−.180	.153	−.117
Teeth-marked tongue	**.258**	**.296**	.080	−.181	.182	**−.279**	−.056	**.254**	**.357**	.217	−.072	−.108	.110	−.187	.112
White tongue coating	.092	−.035	**−.893**	−.074	.052	−.133	.056	.145	.044	.013	−.039	.047	.089	−.067	.079
Yellow tongue coating	−.037	.100	**.895**	−.066	.035	.039	−.094	−.108	.025	−.024	.036	−.076	.088	.108	−.033
Thick tongue coating	.080	**.889**	−.043	.044	−.112	−.016	.002	−.040	−.053	−.021	−.005	.001	−.028	.089	.051
Thin tongue coating	.014	**−.849**	−.095	−.041	.208	−.083	.006	.074	−.093	−.015	.000	−.078	.105	−.096	−.083
Greasy tongue coating	.080	**.685**	.135	−.076	.083	−.127	.160	−.014	.178	.035	.132	−.094	.074	**−.333**	−.134
Wiry pulse	**−.463**	.059	.156	−.007	.168	−.036	.177	.096	−.077	.073	**−.532**	−.051	.046	−.100	−.193
Fine pulse	.085	−.003	−.055	−.121	−.010	.063	.001	.034	−.039	−.127	.139	**.794**	−.008	−.047	−.009
Choppy pulse	.021	.140	−.117	.196	.166	.020	**.450**	−.101	.106	−.129	**−.500**	−.162	.113	**−.323**	−.085
Slippery pulse	−.039	.128	.090	.090	.094	−.055	.051	.121	−.007	.036	**.783**	.110	.070	−.068	−.113

*Note.* Extraction method: principal component analysis. Rotation method: Kaiser standardized orthogonal. Rotation was converged after 20 iterations. Factor load values that were positive and larger or equal to 0.25 are depicted in bold.

**Table 5:** Common factors and their corresponding four diagnostic variables, disease nature, and organ locations.

Common factor	Four diagnostic variables^a^	Syndrome pattern	Organ location of disease
F1	Palpitations (.544); frequent sighing (.629); stomach aversion to cold, listlessness (.388); sensation of coldness of the body and limbs (.795); tinnitus (.395)	*Yang* deficiency	Spleen and stomach
F2	Teeth-marked tongue (.258); thick tongue coating (.889); greasy tongue coating (.685)	Phlegm turbidity	Spleen and stomach
F3	Severe stomach pain (.356); red tongue (.386); yellow tongue coating (.895)	Heat and blood stasis	Stomach
F4	Loose stool (.416); constipation (.502); listlessness (.730); lack of strength (.738); tinnitus (.312); enlarged tongue (.312)	*Qi* deficiency	Spleen
F5	Distention and fullness of the stomach (.332); bitter taste in the mouth (.282); belching (.425); acid regurgitation (.794); heartburn (.702)	*Qi* stagnation	Liver and stomach
F6	Irritability (.590); bitter taste in the mouth (.622); insomnia (.561); profuse dreaming at night (.349); dry mouth (.569)	Heat	Liver
F7	Sharp pain in stomach (.478); tinnitus (.345); dark tongue (.768); tongue with petechiae (.669); choppy pulse (.450);	Blood stasis	Stomach
F8	pale red tongue (.860); enlarged tongue (.251); teeth-marked tongue (.254)	*Qi* deficiency	Spleen
F9	Loose stool (.449); sallow complexion (.775); insomnia (.426); enlarged tongue (.479); tongue with petechiae (.266); teeth-marked tongue (.357)	*Qi* deficiency	Spleen
F10	Distention and fullness in the stomach (.694); constipation (.324); poor appetite (.720); dry mouth (.341)	*Qi *stagnation	Spleen and stomach
F11	Insomnia (.353); slippery pulse (.783)	Phlegm turbidity	Spleen and stomach
F12	Frequent sighing (.251); abdominal distention (.200); belching (.458); nausea (.462); fine pulse (.794)	*Qi *stagnation	Liver and stomach
F13	Constipation (.363); abdominal distention (.295); nausea (.363); profuse dreaming at night (.786)	Phlegm turbidity	Spleen
F14	Palpitation (.759); stifling sensation in the chest (.833); nausea (.395)	Phlegm turbidity	Spleen and stomach
F15	Abdominal distention (.414); fetid mouth odor (.814); enlarged tongue (.293)	Heat and *qi* stagnation	Spleen and stomach

^a^Values in parentheses are results of factor load matrix after rotation transformation.

**Table 6:** Syndrome pattern and their corresponding common factors and four diagnostic variables.

Syndrome pattern		Common factors	Four diagnostic variables
Disease nature	Organ location		
F1 (*qi* deficiency)	Spleen	F4, F8, F9	Loose stool, constipation, listlessness, lack of strength, tinnitus, sallow complexion, insomnia, pale red tongue, enlarged tongue, teeth-marked tongue, tongue with petechiae
F2 (*qi* stagnation)	Liver Stomach	F5, F10, F12	Distention and fullness of the stomach, bitter taste in mouth, belching, acid regurgitation, heartburn, poor appetite, nausea, frequent sighing, abdominal distention, dry mouth, constipation, fine pulse
F3 (blood stasis)	Stomach	F3, F7	Severe stomach pain, tinnitus, dark tongue, tongue with petechiae, choppy pulse, red tongue, yellow tongue coating
F4 (phlegm turbidity)	Spleen Stomach	F2, F11, F13, F14	Constipation, abdominal distention, nausea, stifling sensation in the chest, palpitation, insomnia, profuse dreaming at night, teeth-marked tongue, thick tongue coating, greasy tongue coating, slippery pulse
F5 (heat)	Spleen Liver Stomach	F3, F6, F15	Severe stomach pain, irritability, bitter taste in mouth, insomnia, profuse dreaming at night, abdominal distention, fetid mouth odor, dry mouth, red tongue, yellow tongue coating, enlarged tongue
F6 (yang deficiency)	Spleen Stomach	F1	Stomach aversion to cold, listlessness, sensation of coldness of the body and limbs, palpitation, frequent sighing, tinnitus, teeth-marked tongue

## Abbreviations

CAG: Chronic atrophic gastritis
TCM: Traditional Chinese medicine
EFA: Exploratory factor analysis.
